# The Philosophy of Typhoid or Continued Fever

**Published:** 1868-03

**Authors:** Z. C. McElroy

**Affiliations:** President


### THE PHILOSOPHY OF TYPHOID OR CONTINUED FEVER.—A STUDY OF ITS DYNAMICS AND TREATMENT.

BY Z. C. McELROY, M.D., President.

Read before the Muskingum County (Ohio) Medical Society, Jan. 1, 1868.

In the discussion at our last meeting, on the treatment of typhoid fever, one of our fellows facetiously remarked, that "to treat a case of typhoid fever, it was a needful preliminary to have a case to treat." This remark meant, if it meant anything, that the diagnosis was sometimes difficult, or, if not difficult, the name was given to patients and friends for other purposes.

It occurred to me, that we could better understand a discussion of the treatment of any disease, if we had, as a preliminary, some common understanding of its nature and pathology.

It is my purpose, with the permission of the Society, to present it a picture of typhoid or continued fever, as studied in the light of modern philosophy, and the empirical facts in relation to it, common to medical minds and literature.

And, first, as to its cause or causes—most likely immaterial—the ordinary forces of nature acting in an ordinary way. It may, however, be accepted as certain, that it has two modes of origin, within the body, and from without the body, of those who suffer from it.

These are classed as sporadic, and endemic and epidemic cases. Probably, the most of the cases met with in our locality arise from causes acting within the body of the patient; though cases from contagion—contact with others suffering from it—do occasionally occur.

The symptomatology may be briefly summed up in the words "loss of power, or loss of force." The dynamics of the system are turned into new channels. The stomach, the citadel of the system, refuses or rejects food; or, if retained, passes it on into the bowels undigested, where it undergoes decomposition, giving rise to uneasiness, pain, tympanitis, or diarrhœa. The muscles lose power, patients cannot and do not walk about with elastic step, and perform ordinary labor. The nervous system loses power; the brain cannot and does not act as in health. Every symptom may be referred to loss of power. A patient suffering from typhoid or continued fever must be regarded as in a condition of partial death. Coincident with loss of power, there is loss of bodily weight, whether with or without diarrhœa, sweating, expectoration, or hemorrhage.

With this loss of power and weight, we might, without patient thought, and philosophy to guide us, and the use of that unerring tell-tale of temperature, the thermometer, conclude we either have, or ought to have, diminished heat or temperature; but not so, the "burning disease" was correctly named; the temperature is constantly higher than natural, and its exaltation above the natural standard is a tolerably accurate measure of the intensity of the disease.

Now, what is the meaning of this series of phenomena, philosophically? In endeavoring to find the solution of this inquiry, we must keep constantly before the mind the fact, that no effects of force can be manifest to our senses, except as the result of some preëxisting force, and that no force is ever lost; it may be constantly changing its form, but is never destroyed. Now, why this loss of force and wasting in continued fever? Manifestly, the soft tissues of the body are burning up. And why? Because the fever poison has rendered more or less of them unsuitable for the further uses of the body. The diminished forces of life are diverted into new channels. The dynamics of life are engaged in consuming and removing out of the body the effete or unsuitable tissues, and the phenomena can only be regarded as a conservative process, intended to save life. Boerhave's definition of fever, was, all things considered, the best ever given: that "fever was a mode of life resisting death."

The extent of tissue made useless to the body by the cause or causes of the fever, determines its duration, intensity, and termination.

If all the muscles were in a state of integrity, measured by the standard of health, they would not refuse to act. So of the various tissues of the viscera and nerves, including the sensorium commune.

Just in proportion to the delirium or coma, is the quantity of brain matter unsuitable for the further uses of the organism, large or small.

Nor are the tissues, or parts of tissues, thus rendered useless, if left in the body, harmless, as would be a piece of lead, gold, or silver, accidentally embedded and encysted in the body, nor yet in masses, as in sphacelus, but is deposited or exists interstitially through the muscles and organs, and perhaps enclosed in the interior, or in close proximity to the exterior of the osseous structures, as evidenced by the aching and soreness of the bones, as patients express it.

Of the nature of the changes occurring in the molecular structure of the tissues, from the action of the fever poison, it is impossible to speak, with any certainty, in the existing state of knowledge. We have no means of determining the nature of the changes in the tissues in beings killed by lightning. But the facts are known, that death does occur from shock by lightning. In many instances, there are certainly no changes of which our senses can take cognizance. The inference then, is irresistible, that the electricity has made such changes in the molecular structure of the tissues as to cause death. And the inference is equally irresistible, that the cause or causes of fever induces analogous changes in the various textures of the body, so as to render portions of them unsuitable for the purposes of life.

Further evidence, that such must be the condition of things in continued fever, is the fact that the tissues continue to waste; first, the adipose, then, the muscular and other soft, and, probably, portions of the denser tissues, until the emaciation is sometimes extreme. This waste of the tissues is always a prominent feature in continued fever, commencing sometimes days and weeks prior to the patient taking to bed or seeking medical advice; and this is more especially the case in patients in whom the fever poison is generated in their own systems. Occurring from the poison being taken into the system from without, the prodrome is always shorter, the development of the symptoms more prompt and decisive, and, it may be added, with proper medical treatment, the duration shorter. It may, indeed, prove to be true, that the difference in the origin of continued fevers may constitute the difference between the types known as typhoid and typhus.

Now, the human body in health, probably decays more rapidly than after death. The tissues, in fact, are perpetually renewed during life and health. The dynamics of life are due to the retrograde or destructive metamorphosis of the tissues, just as much as the phenomena of electro-magnetism are due to the oxidation of a metal. The sum of the mechanical, intellectual, emotional, sensational, psychological, and thermal work, or force, of a human body, is an exact measure of the destructive metamorphosis or oxidation of the tissues of the body, and the maintainance of an equilibrium depends on the amount and quality of food that can be taken into the system, and assimilated to its various organs and textures. How long would any of us live without food? And what would be the extent of the waste of the body and the length of life, if compelled to work until nature was exhausted? The time is limited to days, perhaps, scarcely two weeks; and the waste, perhaps, half the weight of the body, or more.

Now, in continued fever, there is a certain amount of muscular, nervous, and vascular tissue rendered unfit for the production of the most of the ordinary results of life and health. If it were not so, we would not have the weakness and waste inseparable from continued fever. The phenomena of the fever are the dynamical results of the oxidation, or destructive metamorphosis, of this destructive tissue. The desire for food has gone, and only those things whose action is known to facilitate destructive metamorphosis are demanded or taken by patients. Of these, water and vegetable acids, as lemon or acetic, are more acceptable than anything else the patient swallows. But no matter how treated by physicians or nurses, patients continue to lose weight—in other words emaciate—day by day, until the effete tissues, or parts of tissues, are oxidized and passed out of the body. The solid debris is found in the urine and alvine discharges; the gaseous products find exit at the lungs and from the bowels; and these gaseous products are offensive according to the amount of nitrogenized tissue undergoing transformation.

Complications arise in the course of the removal of this effete tissue, according as one or several organs have an undue amount of effete matter or tissue interstitialy deposited in them. If in the lungs, it is pneumonia; in the brain, more or less delirium or coma; in the intestines, or, more broadly speaking, abdominal viscera, there will be bowel-complaint, ulcerations, or, perhaps, perforations; in the dermoid tissue, sweating and abrasions; in the bloodvessels, pyæmia; and so on. Now, that is my understanding of continued or typhoid fever. If any fellow of our Society can name a single phenomenon, constant or occasional, occurring in the course of the disease, which cannot be explained by this hypothesis, it will give me pleasure to have it pointed out, for a solitary example has not occurred to my mind.

Viewed thus, the treatment of continued fever becomes a much more simple matter than it can be without this guide from philosophy. No *coup de etats* will be attempted, except in the earliest stages of the disease, originating from causes believed to be outside the body. Under such circumstances, there are very strong reasons for believing that strong emeto-cathartics do sometimes cut the disease short. Under other circumstances, they will fail to accomplish this end, but may prove very advantageous in modifying the intensity of many of the symptoms. No specifics will find much favor in the hands of any physician guided by such views as it has been my endeavor to lay before you. As you cannot safely or profitably interfere with the destructive metamorphosis going on, upon which recovery depends, and giving the products every facility to find exit from the body, attention should be principally directed to supplying the system with the means of reconstructing its tissues. Remembering that the stomach, and, in fact, the whole digestive apparatus, is in a state of partial death, you would not offer it food in such forms as would be acceptable in health, for if you did, and did not wilfully misinterpret the results, you would hardly continue it. The part of digestion supplied by the glands of the mouth are, for the most part, wholly wanting. The capacity of the stomach to assimilate or digest food is fearfully diminished in grave cases. In speaking of symptomatology, you will remember, it was stated that solid food, for the most part, was not and could not be digested, and if not rejected by vomiting, and passed into the intestines, would there add greatly to their already embarrassed and disturbed condition. Food, then, in its highest state of organization, and in its most easily assimilable forms, should be given in small quantities and repeated at very short intervals—there should be no meals. Of food in this form, undoubtedly beef-essence and milk are the most important. Eggs would no doubt occur to your minds as another; but, until the crisis has been passed, eggs are about as likely, if not more likely, to do mischief as good; they decompose too readily, and their decomposition yields too many noxious gases and products, which can only add to the dangers and discomfort of the patient. But after the deepest depression has been reached and the stomach is resuming its tone and power, they are valuable food. Farinaceous articles may be given conjointly with beef-essence and milk, in fluid form, moderately, all through the disease, but the main reliance must be on meat-essence and milk.

If the destructive metamorphosis of unsuitable or effete tissue proceeds too rapidly, the patient succumbs early in the disease, or at whatever stage it takes place. Hence, the cautious use of agents to retard it becomes of the highest importance. The intensity of this metamorphosis is approximately measured by the thermometer, as well as by the circulation and respiration. Hence, ice, spirits, wine, ale, tea, coffee, valerian, belladonna, opium, aconite, and veratrum viride all have their uses, and each, properly timed, confer benefits.

As the life of the patient depends partly on the oxidation of the effete parts of the tissues, and the elimination of the products of the destructive metamorphosis out of the body, it can easily be seen that alcohol is a dangerous remedy, improperly timed, for the reason that its action is known to retard all of the processes going on needful to recovery, *viz.*, assimilation, destructive metamorphosis, and elimination; but exceedingly valuable in the low nervous states characterized by delirium, high temperatures, and excessive elimination

It will, however, more frequently occur that destructive metamorphosis and elimination proceed too slowly, and, as a consequence, digestion and assimilation are well nigh annihilated. For the relief of this condition of things, the mineral acids, especially the chlorohydric, preparations of iron, quinia, chlorate potassa, or bicarbonated alkalies, will be our best agents. The citrate of quinia and iron is a valuable agent, possessing the double property of promoting metamorphosis and elimination, though the perchloride, pyrophosphate, or iron by hydrogen, are very valuable. Sometimes the simple bitters may be advantageously combined with them. Stronger wines and malt liquors are safer and better agents to modify waste than brandy or whiskey.

In the management of the complications, poultices of flaxseed meal, with small proportions of mustard, are of very high value in pneumonia, continuously applied; though it may occasionally be needful to abstract small quantities of blood by leeches or cups, and sometimes apply blisters. And of the brain, blood from the temples, or blisters to the temples or sides of the occiput, with ice to scalp.

For the bowel complaint, better food, that is, food that will not be passed into the intestines undigested, with turpentine stripes, opiate injections to the rectum, opium and alcohol internally; with an occasional necessity for saturnine and vegetable astringents. Abrasions of the surface to be bathed with alcohol, and protected by artificial cuticle of gutta percha in chloroform, or lead plasters.

Viewed in this way, too, the necessity for light, ventilation, cleanliness, and constant attention becomes too obvious to be mistaken or neglected. Frequent sponging or wetting the surface with water or soap and water, or diluted acetic acid, or alkalies, is of the utmost moment.

Such are the outlines of the philosophy of typhoid or continued fever and its remedial management, as it appears to me, studied in the light of the persistence of force, and as cases are managed falling under my especial care. If you can pick them to pieces, do so, for if they are not true, and a safe guide in the management of cases, pray show me the more excellent way.

